# Patient and caregiver experiences of living with dementia in Tanzania

**DOI:** 10.1177/14713012231204784

**Published:** 2023-10-25

**Authors:** Jessica Walker, Catherine Dotchin, Matthew Breckons, Emily Fisher, Godrule Lyimo, Sarah Mkenda, Richard Walker, Sarah Urasa, Jane Rogathi, Aimee Spector

**Affiliations:** Population Health Sciences Institute, 12186 Newcastle University, UK; Population Health Sciences Institute, 12186 Newcastle University, UK; Department of North Tyneside General Hospital, 6072 Northumbria Healthcare NHS Foundation Trust, UK; Population Health Sciences Institute, 12186 Newcastle University, UK; Applied Research Collaboration North East & North Cumbria, UK; Research Department of Clinical, Educational and Health Psychology, 325312 University College London, UK; Department of KCRI, 108094 Kilimanjaro Christian Medical College, Tanzania; Department of KCRI, 108094 Kilimanjaro Christian Medical College, Tanzania; Population Health Sciences Institute, 12186 Newcastle University, UK; Department of North Tyneside General Hospital, 6072 Northumbria Healthcare NHS Foundation Trust, UK; Department of KCRI, 108094 Kilimanjaro Christian Medical College, Tanzania; Department of KCRI, 108094 Kilimanjaro Christian Medical College, Tanzania; Research Department of Clinical, Educational and Health Psychology, 325312 University College London, UK

**Keywords:** Dementia, caregiver, Tanzania, sub-saharan Africa, care needs

## Abstract

**Introduction:** Tanzania is a low-income country with an increasing prevalence of dementia, which provides challenges for the existing healthcare system. People with dementia often don’t receive a formal diagnosis, and with a lack of formal healthcare, are often predominantly supported by family relatives. There are very few published data relating to lived experiences of people with dementia in Tanzania. This study aimed to understand people with dementia, and their caregivers’ experiences of living with dementia in Tanzania and the perceived needs of people with dementia.

**Methods:** Qualitative, semi-structured interviews were conducted with 14 people with dementia and 12 caregivers in Moshi, Tanzania. Interviews were audio-recorded, translated, transcribed and analysed using a Framework Analysis approach.

**Results:** Three sub-themes were identified within data describing the experience of ‘Living with Dementia in Tanzania’: ‘Deteriorations in Health’, ‘Challenges to living with Dementia in Tanzanian Culture’, and ‘Lack of Support’: people with dementia faced challenges due to social isolation, stigmatisation, and lack of caregiver knowledge on how best to provide support. Collectively, these impacted on both the physical and mental health of people with dementia. Misconceptions about dementia aetiology related to age, stresses of daily life and other co-morbidities. People with dementia were motivated to access treatment, exhibiting pluralistic health-seeking behaviours. There was an overall preference for non-pharmacological interventions over medication, with high levels of trust in medical professional opinions.

**Conclusions:** Living with dementia in Tanzania is influenced by both cultural and religious factors. More work is needed to target supplementary healthcare (with efforts to promote accessibility), support for caregivers and public health education about dementia to overcome existent misconceptions and stigma.

## Introduction

Worldwide, an estimated 55 million people live with dementia, with 10 million new cases diagnosed each year (World Health Organisation, 2022). Around 27.3 million of people with dementia are from low- and middle-income countries (LMICs) (Prince et al., 2015), accounting for 58% of people with dementia worldwide (Stoner et al., 2021). Dementia poses a significant global health burden, with figures in LMICs forecast to increase substantially over the next 30 years (Prince et al., 2015). In Tanzania, in the over 70 years age group, dementia is almost three times more common in women than men (Longdon et al., 2013). Dementia in Tanzania is associated with excess mortality relative to those with no cognitive impairment (Paddick et al., 2015b), similar to other LMICs (Katzman et al., 1994; Nitrini et al., 2005; Wang et al., 2010).

Currently, there is no national programme to tackle dementia in Tanzania (Mushi et al., 2014), with health facilities struggling to meet needs of people with cognitive impairment (Mushi et al., 2010, 2012). The burden of co-morbidities (commonly undiagnosed) for people with dementia complicates management of dementia (Clausen et al., 2005; Dewhurst et al., 2012). While medications and psychosocial interventions form first line treatments in HICs, scarce resources and limited specialist care in LMICs provide challenges for both diagnosis and management of dementia (Dotchin et al., 2013). A study by Mushi et al. (2014), found that nearly half of people with dementia and their caregivers had overall positive views of modern healthcare (Mushi et al., 2014). However, the majority also reported a lack of perceived improvements, similar to alternative treatments accessed (such as traditional healing and faith healing) (Mushi et al., 2014).

Limited access to transport, and financial hardship, exacerbate the challenge of accessing medication (Paddick et al., 2015b). Mushi outlined that many people with dementia in the Hai District (rural Tanzania) had no pension or health insurance, meaning any medical support needed to be paid for out of pocket (Mushi et al., 2014). Most people in this study were poor, widowed and economically inactive posing significant financial challenges for accessing treatment (Mushi et al., 2014).

Much of the support for people with dementia comes from unpaid relatives, who lack knowledge about the condition and have not been given any specific training or skills in this area (World Health Organisation & Alzheimer’s Disease International, 2012). Caregivers of people with dementia in Tanzania do not have the support of professional counselling, support groups and healthcare infrastructure, as in HICs, that is needed to improve their caregiver abilities and quality of life outcomes for the people with dementia (Clarke et al., 2013; Lopez-Hartmann et al., 2012).

Public education about the condition, dementia training for health professionals and social support have been identified as urgent priorities (Larweh, 2021). Ageism, reflecting a widespread negative view of ageing, is a problem admitted by Tanzania’s government (Ministry of Labour Youth Development and Sports, 2003), seeing elderly people as “dependent” rather than “productive” members of society (Angus & Reeve, 2006). This may be hindering achievement of health inequality goals (Lloyd-Sherlock et al., 2016).

Cultural emphasis on discouraging older people from carrying out complex tasks may contribute to worsening cognitive function, predisposing to poor outcomes (Paddick et al., 2013). Spirituality and superstitions continue to affect health behaviours (Brooke & Ojo, 2020; Larweh, 2021). For example, dementia is seen by some as “God’s will” or a punishment (Mukadam et al., 2011). Poor knowledge of the condition relates aetiology to natural processes of ageing (Larweh, 2021; Mushi et al., 2014), with no equivalent term for dementia in local languages (Wimo et al., 2017). It is commonly referred to as “ugonjwa wa uzeeni” (disease of old people) or “memory loss disease” (Mushi et al., 2014).

Research exploring experiences of living with dementia in Tanzania has so far focused on those newly diagnosed with dementia (Mushi et al., 2014). This study aimed to understand people with dementia and their caregivers’ experiences, identify challenges of living with dementia in Tanzania and explore perceived support needs.

## Methods

### Study setting

This was a qualitative study, consisting of interviews with people with dementia and their caregivers. People with dementia were recruited from a Cognitive Simulation Therapy (CST) programme, alongside their caregivers. CST is a non-pharmacological intervention for dementia, developed in the United Kingdom (Spector et al., 2003) and adapted for use in Tanzania (Mkenda et al., 2018). These CST groups were part of a wider CST-International project, exploring implementation of CST in Tanzania, with further information on screening, recruitment and running of groups detailed previously (Spector et al., 2019).

Interviews took place in the psychiatry department at Kilimanjaro Christian Medical Centre (KCMC), a tertiary referral hospital in Moshi, Northern Tanzania. The national language is Swahili, and there is a large emphasis on agriculture, with most of the population working as farmers, relying heavily on growing crops, such as tomatoes and coffee (Paddick et al., 2017). The main local tribes include Chagga, Maasai, Sambaa and Pare. Religion forms an important part of people’s lives, with most identifying as either Christian or Muslim.

### Participants

#### People with dementia

The inclusion criteria for people with dementia were as follows: (a) completion of a CST programme within the last month, (b) meeting International Classification of Diseases 10 (ICD-10) criteria for mild to moderate dementia (World Health Organisation, 1993) (confirmed by a doctor with psychiatry training), having had initial screening for dementia using the IDEA tool, which has been developed and validated in Tanzania (Paddick et al., 2015a) (c) sufficient verbal communication to engage in an interview, and (d) capacity to provide verbal and written consent (‘signing’ with a thumbprint if unable to write) or assent from a caregiver if lacking capacity.

#### Caregivers

The inclusion criteria for caregivers were as follows: (a) age 18 years and over, (b) unpaid caring responsibilities for a participant of CST, and (c) able to provide verbal and written consent.

Sample size for the people with dementia and caregiver group was guided by principles of Information Power (Malterud et al., 2016), as well as pragmatic considerations such as time in the field.

#### Interview procedure

Semi-structured interview guides were informed by previous work (Morrish et al., 2022), with one developed for people with dementia and another for caregivers, focussing on experiences of living with dementia. Interview guides were refined throughout the interviewing process to clarify questions as necessary or further explore topics arising in previous interviews.

Guides were written in English, translated into Swahili and back translated by a second translator, as recommended in previous research (Sousa & Rojjanasrirat, 2011; World Health Organisation, 2018), to ensure semantic equivalence between the original and translated guides (supported by Brislin, 1970; Chapman & Carter, 1979). Translation was carried out by two local members of the research team, who were experienced in qualitative research and translation work, and fluent in Swahili and English.

Interviews took place in a quiet, private room to minimise disruption. Interviews were led by (one of) two translators with extensive experience in qualitative interviews, using an encrypted dictaphone (Philips DPM8100 Digital PocketMemo) to record interviews. They were both independent of the CST team and any patient care, with no previous involvement in research relating to CST, limiting bias. Interviews were undertaken in Swahili, with responses summarised in English to allow opportunity for additional questions from the researcher. The presence of a white, female researcher in interviews was conspicuous and may have impacted responses to questions (Teman & Richard, 2017). To minimise impact from this, the interviewer explained the researcher’s role before interviews and the researcher was positioned in a corner of the room to demonstrate an observer role.

Demographic data for participants and caregivers were collected prior to interviews and included transport (method, cost, supported or independent), co-morbidities, medications, duration of dementia symptoms, and whether participants had health insurance.

#### Consent

Information sheets and consent forms were verbally explained to ensure those who could not read or understand the information fully were informed. There was an opportunity to ask the research team questions before providing consent. Capacity to consent was assessed by the interviewer. All participants provided written consent for interviews.

#### Transcription process

Transcriptions were completed and translated by researchers with experience of qualitative research who were fluent in English and Swahili. Translations were checked by the lead author and clarifications were made with the original translator. Age and status-appropriate pseudonyms (informed by Lewis et al., 2018) were used to maintain anonymity of participants.

#### Data analysis

Data analysis followed a five-step Framework analysis process, described by Ritchie and Spencer (Ritchie & Spencer, 1994): (1) familiarisation with transcripts, (2) identifying key concepts and themes to form the basis of a thematic framework (later refined throughout the analysis stage), (3) indexing (coding) data (using NVivo data management software version 12 (NVivo Qualitative Data Analysis Software Ltd, 2020), (4) charting indexed data into clearly defined themes and sub-themes to form a working analytical framework, and (5) mapping and interpretation, involving exploration of relationships between themes (Gale et al., 2013; Gubrium & Holstein, 1997).

With a personal role in the research (Jackson et al., 2007), findings were discussed and justified with other researchers over the course of the project. Reflexive fieldnotes were kept throughout data collection and analysis to contextualise the data and develop findings, as recommended by Stocking (Stocking, 1983).

## Results

### Sample

All 14 people with dementia participating in the two CST groups at KCMC, met inclusion criteria for interviews and were recruited. Two participants were interviewed with their caregivers to support communication. Participant interviews lasted between 24 and 51 minutes.

Twelve caregivers were interviewed (one participant was independent (with no identified caregiver) and one caregiver declined participation). Caregiver interviews lasted between 23 and 45 minutes.

Demographic information for people with dementia and caregivers is summarised in Table 1.

**Table 1.** Demographic data for people with dementia and caregivers interviewed.

	People with dementia (*n* = 14)	Caregivers (*n* = 12)
Age (years)		
<60	1	5
60–69	3	4
70–79	7	3
80+	3	0
Average age (±SD)	72.6 (±9.0)	61.6 (±10.1)
Sex		
Male	9	4
Female	5	8
Marital status		
Married	10	10
Single	1	1
Divorced	1	1
Widowed	2	0
Education (highest level)		
Never attended	2	0
Completed primary	6	5
Completed secondary	5	4
Completed tertiary	1	3
Religion		
Christian	13	—
Muslim	1	—
Tribe		
Chagga	12	—
Sambaa	1	—
Pare	1	—
Primary language		
Swahili	13	—
Chagga	1	—
Employment		
Professional (health, teaching)	—	2
Agriculture	—	6
Small business	—	3
Retired	—	1
Relationship to participant		
Spouse	—	6
Sibling	—	3
Child	—	2
Neighbour	—	1

*n*, number; SD, standard deviation.

Chagga, local tribal language; Swahili, national language.

Participants interviewed were living with a mean of 2.6 diagnosed co-morbidities in addition to dementia, and were on a mean of 2.7 different medications. The most common co-morbidities were diabetes, hypertension, cardiac problems, and depression, with medication use predominantly for diabetes and hypertension. All but one participant was on a National Health Insurance Funded plan. National insurance covers those who are working, or have previously worked, as public employees, students and those under 18 years of age.

Participants came from a range of highland, midland, and lowland areas to reach KCMC for the intervention. “Highland” villages (further up the mountainside) are generally related to higher economic status, with “lowland” villages (further down the slopes) comprising some of the more deprived areas of the population. Most participants used public transport to reach KCMC, where the intervention was based, using multiple forms of transport to travel long distances with long journey times (mean 2.8 hours return journey). The mean reported cost of the return journey was $5.92.

Most caregivers (8/12) lived with the people with dementia they were supporting. Care time needed was most commonly reported between 3 and 4 hours per day by caregivers. The majority of people with dementia had more than one caregiver.

## Themes

**Figure 1.**
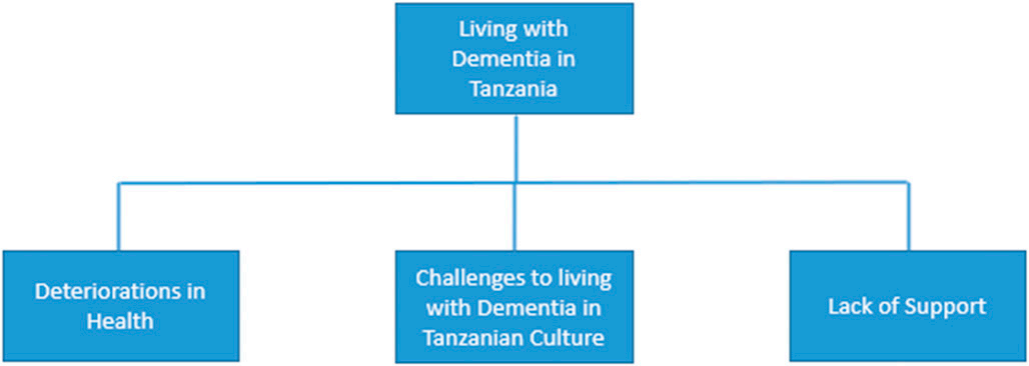
Overarching theme and sub-themes.

Within the main theme ‘Living with Dementia in Tanzania’, three sub-themes were identified; ‘Deteriorations in Health’, ‘Challenges to living with Dementia in Tanzanian Culture’, and ‘Lack of Support’ (shown in Figure 1). Quotes are included to illustrate data and support themes, followed by participant/caregiver status, sex, and age to contextualise data.

### Deteriorations in Health

Description of symptoms varied between participants, with mean duration of symptoms relating to dementia being three years. Both physical and mental health symptoms were reported;

“C14: She use to say I feel bad, I am tired, the body is weak, I do not know what to do, you know someone lose hope of doing the job because of thoughts.” (caregiver, 41 years, female)

Male participants seemed to report more struggles with increased anger and frustration, whereas female participants seemed to experience more feelings of sadness and loneliness.

Most participants were unable to complete usual tasks around the home;

“P1: My years have lost direction totally. Now it is more than two years since I cannot go in the kitchen and cook.” (person with dementia, 86 years, female)

These were dramatic changes from the way that participants had been before. Some individuals struggled to remember names, whilst others had become frustrated with themselves and others at home when misplacing items.

One particularly distressing symptom experienced was vivid visual hallucinations;

“P7: At night I scream and see people holding my neck like robbers. I scream so much to the extent that other people wake up.” (person with dementia, 86 years, male)

Many people with dementia felt socially isolated; this could be exacerbated by life events such as the loss of a loved one, retirement or the removal of home duties in line with Tanzanian culture.

People with dementia recognised the importance of socialising as a significant contributor to overall wellbeing;

“P13: you become happy sometimes because you are with others, so you be happy, you laugh and exchange ideas..., and at that time the thoughts stop for a while.” (person with dementia, 71 years, male)

However, due to deterioration in cognition, opportunities for socialising in communities, such as religious gatherings, were sometimes missed.

Many participants and caregivers knew multiple people from their local area with similar cognition and memory problems, claiming it affected many people;

“P1: There are many at their homes. They cannot even get up, they are just like me, my neighbours, there are many.” (person with dementia, 86 years, female)

## Challenges to living with dementia in Tanzanian culture

People were responsive to different wording used to describe dementia. With no direct translation for dementia (Wimo et al., 2017), both “shida ya akili” and “kupoteza kumbukumbu” were used throughout interviews. However, “shida ya akili” evoked quite strong reactions from some caregivers as this wording is more linked to mental problems and “craziness”, whereas “kupoteza kumbukumbu” was more accepted, relating to memory loss and closer to the definition of dementia.

Caregivers illustrated misconceptions of dementia aetiology relating to age, stresses and loneliness (social withdrawal). Caregivers were accepting of dementia as a normal process of ageing and not as a medical problem (referring to it as ‘old people disease’);

“C11: I used to think it’s because of ageing and stress. After his wife passed away, he got so much stress. He started forgetting.” (caregiver, 72 years, male)

One caregiver saw dementia as a manifestation of psychological stress from personal family life;

“C12: People have many family problems and they can’t expose it so it is their secret. So when it is a secret it is like a burden so it then affect them psychologically. But when they have a chance to tell it to someone such as you and be cancelled you find out their life recovers normal. They can overcome the stress and their life be easier.” (caregiver, 55 years, female)

Limited knowledge of dementia meant that some were unaware of their possible dementia status;

“C3: There are others with such conditions, they have not noticed, are not aware or their relatives have not noticed.” (caregiver, 61 years, male)

Stigma was reported in local communities for those with memory problems. Psychiatric conditions were less likely to be recognised or accepted as requiring medical help. One caregiver suspected witchcraft as the cause of memory problems for others in their local community;

“C11: I know about three of them. I believe somehow in their family it’s due to witchcraft that these people are losing memory.” (caregiver, 72 years, male)

While most of the participants suggested that there was little understanding about dementia, one participant described feeling that dementia was an accepted condition that can affect anyone, and was supported by friends experiencing similar problems;

“P10: They told me you just go, if things will be good, we will also come.” (person with dementia, 70 years, male)

Manifestation of symptoms of dementia was not well received in local communities, which could lead to social exclusion;

“C12: Some get lost, others become harsh and talk a lot, something which is not good to the community that they live. It becomes a disturbance.” (caregiver, 55 years, female)

“P13: someone has dementia but they use to say this one has become elderly, ignore them. They don’t know what they are doing.” (person with dementia, 71 years, male)

This social exclusion was also described in the home setting, with a culture of removing responsibilities from participants due to age and co-morbidities, resulting in unstimulating, lonely environments. Care generally fell to family relatives, who reported it as their responsibility. Caregivers sometimes found these duties of care overwhelming;

“C13: The responsibilities can’t be less as I have to help him, as he can’t wash cloth, he cannot go in the kitchen and cook so I have to cook, I have to do wash the cloth.” (caregiver, 62 years, female)

The few that had tried to alleviate their symptoms had first tried herbal remedies (primarily rosemary) and faith healers before resorting to formal medical care;

“C10: He used to try eating local herbs and foods to help him get back his memory, but I haven’t seen it helping him.” (caregiver, 56 years, female)

## Lack of support

Although people described accessing medical help for other conditions, healthcare access for dementia was limited. Only one participant had undergone a computed tomography scan of the brain to aid assessment of his cognition.

There was an overall preference for a non-pharmacological intervention over medication, emphasised by a caregiver supporting someone with complex co-morbidities requiring multiple types of medication;

“C7: Already he is using a lot of medication. I wouldn’t like that (access to medication) to happen.” (caregiver, 78 years, female)

There was a general lack of confidence that medication would have the desired outcomes and concerns about potential side effects;

“P12: I do not want medicine because my problem is not that big. Medicine has got side effects. Chemicals are not good for our health.” (person with dementia, 75 years, female)

However, people with dementia and their caregivers discussed the trustworthiness of doctors extensively, with any treatment recommended perceived to be the best option.

Reimbursement for travel journeys to attend interviews was seen to be valuable, helping those that would otherwise be unable to afford it;

“C7: The transportation helped a lot because there are times you wish to attend but you have no transport but since they offered it, he used to attend always.” (caregiver, 78 years, female)

Participants emphasised a need for the provision of more local health services to promote accessibility for health appointments and interventions, for instance in the format of mobile clinics. They also emphasised the importance of maintaining neutral (non-religious) environments for healthcare.

Participants thought that more efforts from the government needed to go into identifying dementia earlier;

“P12: When you have a problem, you have to take a quick reaction to solve the problem before it becomes a big problem. So, treating a problem at early stages makes it easy for one to recover.” (person with dementia, 75 years, female)

One caregiver highlighted a need to consider ways of preventing dementia, with so many individuals thought to be affected;

“C12: A big percentage of the elders get that problem of dementia. Others get mad and confused so I think it is better to prevent than to treat.” (caregiver, 55 years, female)

Public health education for dementia was deemed essential to tackle misconceptions about dementia, enabling people to engage with treatments;

“C5: If people are not educated they won’t know, they will think that it is age that has made them forget. People are lacking education and don’t understand the behaviour of forgetting a lot is a disease.” (caregiver, 66 years, female)

Some caregivers thought that education was also needed to know how best to support the people with dementia. Suggestions for the best platform to educate and reach the most people included government announcements on the radio and television, churches and mosques. People felt that funding of public health education and provision of additional healthcare services could be supported by government schemes.

## Discussion

### Sample demographics

Although dementia is estimated to be more common in women in Tanzania (Longdon et al., 2013; Morrish et al., 2022; Mushi et al., 2014), the majority of our sample was male (9/14). Female participants were also predominantly younger than the men. Male participants perceived more females to be affected in communities but can be “shy” and do not have the same means and availability to access healthcare services, with additional home responsibilities as per Tanzanian culture (Feinstein et al., 2011). This may explain the lack of female dementia participants if they were less likely to be accessing healthcare and therefore were less likely to be recruited, with recruitment based in hospital.

### Symptoms

Dementia symptoms were generally longstanding and had progressively worsened slowly over time. Misplacement of items, forgetting people, and communication difficulties were commonly described, similar to previous findings (Guerchet et al., 2017; Mushi et al., 2014).

Male participants seemed to exhibit aggressive and regressive behaviours, with depressive symptoms more common in women. While our research methods and small sample size do not enable correlation to be examined, patterns in our data suggest there may be similarities in this sample with previous findings (Lövheim et al., 2009). The variety of symptoms seen widely among people with dementia (Kales et al., 2015; Kansagara & Freeman, 2010) may limit success of future interventions for dementia and create challenges for accurate diagnosis due to the range of presentations.

### Knowledge and beliefs about dementia

Acknowledgement of symptoms relating to dementia was poor, instead attributing these to natural biological processes of ageing, general stresses of daily life, loneliness or other co-morbidities as previously described (Guerchet et al., 2017; Larweh, 2021; Mushi et al., 2014; World Health Organisation & Alzheimer’s Disease International, 2012). Some people with dementia were open about their condition, supporting findings that dementia perhaps does not carry the same burden of stigma as other chronic conditions (Ineichen, 2000; Mshana et al., 2011; Mushi et al., 2010; Zeilig, 2014). However, the people with dementia interviewed were a select group who had presented to hospital and then agreed to be recruited. Hence, this openness about dementia may not be representative of the overall population of people with dementia in Tanzania.

There was still some evident stigma, relating dementia aetiology to witchcraft, which has also been described in previous work (Stroeken, 2002). Overall, these beliefs and superstitions can act as barriers to families identifying dementia symptoms in their relatives and accessing appropriate support for them, contrary to HICs where education and support is much more accessible.

### Education

Suggestions for public health education, using a range of media platforms, to tackle these misconceptions and help the many unknowingly affected (with support from government schemes) reinforces previous findings (Adedeji et al., 2022; Larweh, 2021; Oliver, 2020). However, with only 40% of the population accessing electricity (The World Bank, 2022a), suggestions for radio and television announcements may not be able to reach all those affected. Suggestions for announcements at places of worship and for village leaders to identify affected people with dementia may be more appropriate in this setting. Education on dementia and preventative strategies may help to identify and manage dementia earlier, slowing overall deterioration of symptoms and possibly delaying the need for high-cost care (World Health Organisation & Alzheimer’s Disease International, 2012). The perceived trustworthiness of doctors suggests information may be better accepted from medical professionals. However, the sample interviewed were already accessing medical support, so it is unsurprising that they were in support of medical professionals’ advice.

Alterations of wording used for dementia evoked strong reactions for some of those interviewed. Within Tanzanian culture, language used is sometimes not very reflective of reality, with polite terms used to describe difficult or unacceptable things. This makes it challenging to live with dementia in this context, with no formal identification of dementia as a word in Swahili, and language to describe it ambiguous in its true meaning. Choice of wording would need careful consideration for public education on dementia to promote appropriate health seeking behaviours, avoiding phrases that may contribute to stigma or cause offense.

### Social support

A culture of care was identified, with reliance on relatives (often multiple) to support people with dementia, in accordance with previous findings (World Health Organisation & Alzheimer’s Disease International, 2012). Most caregivers (8/12) lived with the people with dementia, perhaps due to this dependence for support. This culture of care is likely to be linked to lack of formal care availability as for other chronic diseases in Tanzania (Meena, 2010). Caregivers took away responsibilities from people with dementia, showing “malignant social psychology” where people with dementia are devalued (Kitwood, 2002), reinforcing ageist attitudes and potentially predisposing to poor outcomes, as described previously (Paddick et al., 2013). Caregivers consequently felt overwhelmed, lacking knowledge and skills in how to care for people with dementia as previously seen (Adedeji et al., 2022), unlike support existing in HICs (Clarke et al., 2013; Lopez-Hartmann et al., 2012). This may have predisposed caregivers to social isolation, as described by Brodaty and Luscombe (Brodaty & Luscombe, 1998).

The wider impact of dementia on families, reinforces the importance of including both participant and caregiver perspectives to understand how best to support affected families, as emphasised by the World Health Organisation (WHO) and Alzheimer’s Disease International (World Health Organisation & Alzheimer’s Disease International, 2012). Ways of providing social support outside of families will become increasingly important as increasing migration from rural to urban areas in Sub-Saharan Africa, particularly for young women, will lead to a decline in availability of family support systems for people with dementia (Tanyi et al., 2018; Uwakwe et al., 2009). Effective coordination will be needed between modern care and informal health care to best support people with dementia, as described previously (Mushi et al., 2014).

People with dementia expressed a need for increased socialisation due to lonely, unstimulating home environments and missing out on social interaction. They were ignored due to beliefs that they were “crazy”, suffering with a mental health disorder and hence viewed as not fitting in with society, therefore ‘othered’. The importance of maintaining good levels of socialisation has been highlighted by the WHO and Alzheimer’s Disease International (World Health Organisation & Alzheimer’s Disease International, 2012). Group interventions or support groups can be beneficial for people with dementia to meet others experiencing similar difficulties, whilst reducing the burden of care on the family (Grässel et al., 2010). It could also enable caregivers to have the opportunity to engage in more productive work (Mauritius Ministry of Social Security, 2010). Other suggestions related to education for caregivers about how to care for people with dementia. This has been identified in previous research as an urgent need to improve existing care for people with dementia (Larweh, 2021).

### Healthcare Support

People with dementia in this study considered accessing treatment a high priority, for some on par with religious commitments, to improve their own health and reduce the burden on family caregivers. There is a large emphasis on alternative herbal treatments and contact with faith healers within Tanzanian culture when approaching health problems. These were mostly perceived to be unsuccessful, in line with previous research, which found more positive attitudes towards formal healthcare (although these often related to treatments for other health conditions rather than for dementia) (Mushi et al., 2014).

Another aspect of care for people with dementia relates to management of co-morbidities, seen as a burden to participants in this study, although people with dementia did not report any stigma experienced from accessing support for other health conditions. People with dementia often live with more chronic conditions than their age counterparts without dementia (Schubert et al., 2006), with chronic conditions such as type two diabetes and cardiovascular disease having the potential to affect progressive neurodegeneration (Edwards et al., 2019). Management often becomes more difficult as dementia symptoms progress (Bunn et al., 2014) and goals of care change (Taghizadeh Larsson & Österholm, 2014). More work is therefore needed to support access to ongoing management of co-morbidities for people with dementia in Tanzania, with the majority of people with dementia in Tanzania without healthcare insurance (Mushi et al., 2014) currently unable to afford treatment, contrary to our sample.

Due to this burden of co-morbidities, most people with dementia were keen for a non-pharmacological intervention for dementia. Medication introduced worries about accessibility and cost, alongside fears of harmful side effects and negative views that medication may not have the desired outcomes. However, interview responses may have been influenced by recent completion of a non-pharmacological intervention, serving as the only formal healthcare support received for many participants. Only those that had accepted the intervention were interviewed, and views of those who declined the intervention may have been more favourable towards medication.

Participants travelled to their local tertiary hospital for interviews. Difficulties identified with this venue related to complex, expensive travel journeys. These were deemed to be unaffordable for some if not reimbursed for travel, particularly in light of high fuel prices at the time of the study. Return travel journeys cost $5.92 on average, which is considerable with average income per person per day only $3.11 and almost half (49.4%) of the population living below the poverty level ($1.90 a day) (The World Bank, 2022b). Unaffordable travel costs in addition to many people with dementia lacking a pension or health insurance (Ajwad et al., 2018; Mushi et al., 2014) highlights the poor accessibility of healthcare, adding to the overall challenge of living with, and managing, dementia. Future efforts within formal healthcare will need to consider ways of improving this accessibility.

Participants additionally noted that there are many people suspected of having dementia who are unable to leave their homes. When considering locations for healthcare appropriate for people with dementia’s needs, suggestions were made for mobile health clinics alongside tertiary hospital support. Previous research has identified the possibility of partnering with local churches and mosques, who already deal with social problems in communities, regardless of religious backgrounds (Mushi et al., 2014). However, participants in this study considered it important to maintain a neutral setting, to ensure individuals were not segregated and were treated as equals. This is supported by CST research concluding that church settings may lead to alienation of faiths (Oliver, 2020; Paddick et al., 2017), and limit depth of conversations (Mkenda et al., 2018).

### Strengths

This is the first study to explore experiences of people living with dementia in Tanzania participating in a psychosocial intervention. The semi-structured interviews allowed in-depth exploration of participants’ lived experience. All 14 people with dementia who took part in the psychosocial intervention, and all but two caregivers, consented to a qualitative interview, which reduces selection bias from the initial sample. Additionally, the mean age of people with dementia in our sample (72.6 years) and predominance of Chagga tribe origin was comparable to a recent study showing an increasing prevalence of dementia over the last 10 years in the Kilimanjaro region (Yoseph et al., 2021).

We undertook a robust and systematic analysis of data using recognised methods, being rigorous in querying data. Interviews of both people with dementia and caregivers gave a comprehensive overview of the people with dementia and their lived experiences of dementia.

### Limitations

The sample may not reflect widespread experiences of living with dementia due to some demographic differences between this sample of people with dementia and the wider Tanzanian population. The Muslim community was underrepresented (1/14) compared to roughly a third of the Tanzanian population identifying as Muslim (Office of International Religious Freedom, 2022). Additionally, the majority of the sample was male (9/14), whereas dementia is estimated to be more common in women in Tanzania (Longdon et al., 2013; Morrish et al., 2022; Mushi et al., 2014).

The education level and socioeconomic status of people with dementia was generally higher than previous research in Tanzania, where most people with dementia had no primary education and the main occupation was farming (Mushi et al., 2014). In our sample, some participants had professional jobs, such as teaching, and all but one had national health insurance (Mushi et al., 2014). This higher education level and socioeconomic status is likely due to recruitment from a secondary care setting. However, the financial strain created by the cost of transport to access healthcare in our sample, in addition to the cost of services for those without insurance, is likely to be similar in the general population in Tanzania.

As per the aim of qualitative research, we sought to gain in-depth insight into the experience of a group of participants. These participants were recruited from CST groups through convenience sampling, so their experiences may not be reflective of the many people with dementia and caregivers in Tanzania who are not engaged in psychosocial interventions. Future research should expand recruitment strategies to explore a broader range of experiences across a larger sample.

The main role of the white, female researcher was to organise the research and oversee the interview process while not being directly involved with the interviews. Her presence during the interviews may have influenced participant responses, for example by causing the participants to feel inhibited or observed. To minimize this, the researcher’s presence was explained to participants and it was made clear that she did not understand Swahili.

It was challenging to obtain complete semantic equivalence between translated transcripts and original versions. Lack of translator’s direct experience in healthcare for dementia may have affected the quality of translation (Birbili, 2000), however discussions clarifying translations of transcriptions aimed to minimise any misrepresentations of the data.

Analysis of qualitative data is subjective, and the researcher being based in a very different culture means interpretation of the data may differ from that of someone living in Tanzania, as it is filtered through individual experiences (Temple & Young, 2004). However, recognising this personal role in the research (Jackson et al., 2007) and justifying findings to other researchers over the course of the project has improved the trustworthiness of data collation, with impact further minimised through discussion with translators and local researchers about the data.

### Conclusions and Recommendations

Living with dementia in Tanzania is complex, and influenced by both cultural and religious factors. People with dementia face challenges in communities due to social isolation, stigmatisation and lack of caregiver knowledge on how best to support people with dementia. Although knowledge of symptoms is generally sufficient, there are misconceptions about dementia aetiology relating to age, stresses of daily life and other co-morbidities.

People with dementia exhibit pluralistic health-seeking behaviours, however there were overall positive views about formal healthcare, with a high level of trust in medical professionals. People with dementia were motivated to access treatment for control of symptoms and to reduce burden of care on families.

There is an urgent need to target supplementary healthcare, support for caregivers (such as supplementary social support) and public health education about dementia at a national level. Funding support (such as government schemes) needs to be established to enable accessible future support. More work assessing the costs of healthcare, and costs of travel for this, would help inform future decisions on strategies to promote accessibility of healthcare. Alongside this, more work is needed to promote equality of access to healthcare. Education should detail dementia aetiology, symptoms and importance of accessing treatment. This is important to alter socially-constructed perceptions which will impact on future intervention effectiveness.
